# In Vitro and In Vivo Effect of Poplar Bud Extracts on *Phytophthora infestans*: A New Effective Biological Method in Potato Late Blight Control

**DOI:** 10.3390/plants9020217

**Published:** 2020-02-08

**Authors:** Botond Turóczi, József Bakonyi, Károly-Attila Szabó, János Bálint, István Máthé, Szabolcs Lányi, Adalbert Balog

**Affiliations:** 1 Department of Bioengineering, Faculty of Technical and Social Sciences, Sapientia Hungarian University of Transylvania, Libertății Square 1, 530104 Miercurea Ciuc, Romania; 2 Deparment of Organic Chemistry, Faculty of Applied Chemistry and Materials Science, Polytechnic University of Bucharest, Splaiul Independenţei str. 313, Sector 6, 060042 Bucharest, Romania; matheistvan@uni.sapientia.ro (I.M.); lanyiszabolcs@uni.sapientia.ro (S.L.); 3 Department of Plant Pathology, Plant Protection Institute, Centre for Agricultural Research, Herman Otto str. 15, 1022 Budapest, Hungary; bakonyi.jozsef@agrar.mta.hu; 4 Department of Horticulture, Faculty of Technical and Human Sciences, Sapientia Hungarian University of Transylvania, Sighişoarei 1/C, 540485 Tîrgu-Mureş, Romania; szabo_ata@yahoo.com (K.-A.S.); balintjanos@ms.sapientia.ro (J.B.)

**Keywords:** biological control, poplar bud extract, conventional treatment, field application, organic farming, resistance

## Abstract

The effect of populin extract from black poplar (*Populus nigra*) on seven different late blight strains was tested under laboratory and field conditions. The growth rate of hyphae was found to be significantly lower in vitro after 3 and 4 *v*/*v*% populin applications. Stain M16 was resistant to populin treatment under lab conditions, however. Both 5% and 10% concentration populin reduced the M16 strain’s severity on potato leaves under field conditions and proved to be even more effective than conventionally used fungicides Infinito 687 Sc and Valis M. Higher infection intensity at the 1% level was observed after 24 h using Valis M, and the same trend toward 10% infection remained after 48 and 72 h as well. Low, almost-no-infection intensity was detected after populin 5% and 10% treatment under an open field condition. Altogether, it can be concluded that populin extract can be a low-cost option for growers and an environmentally friendly approach in late blight control.

## 1. Introduction

Late blight caused by *Phytophthora infestans* (Mont.) de Bary is one of the most severe and economically important diseases of the *Solanaceae* family worldwide [1]. It was responsible for the disastrous Irish potato famine in the 1850s, during which millions of people, especially children, starved and even died. Millions more were forced to emigrate [2]. The entire host plant can be severely infested very quickly [2,3]. In the past few decades, the disease has re-emerged in several parts of the world as a more aggressive pathogen of potato and tomato [4,5].

Some management methods have been developed to control late blight. Because late blight can survive not only on the living tissue of plants but in soil too, transplants or imported potato tubers or tomatoes are the most significant sources of early infestation. Therefore, one of the most effective management strategies is to avoid sources of early season inoculum [3]. Conventionally, (commercially available) fungicides are largely used to control late blight; however, several resistant strains against these fungicides have been reported [6,7,8]. Resistance to metalaxyl and other fenilamids is already well known from The Netherlands [6]. Also the use of copper-containing formulations has precipitated an urgent need for alternative control methods all over in EU [9].

Using different natural products such as plant extracts is considered an alternative method and there has been increased interest in developing treatment strategies based especially on natural plant products [10,11,12]. Under laboratory conditions, the effects of allicin (extracts from garlic) were tested on vegetative mycelial growth [13]. The allicin application significantly reduced the colonization of potato tubers. The inhibitory effects of allicin on the germination of sporangia and encysted zoospores as well as the subsequent reduction in germ tube growth have also been reported [14]. Other plant extracts may have similar effects, with one potential compound being populin, an extract from black poplar (*Populus nigra*). The positive effects of populin against apple scab have been reported under both lab and field conditions [15,16,17]. Because potatoes represent one of the most economically important vegetables, and because late blight resistance against conventional fungicides has already been demonstrated, we considered that populin may have a similar effect on late blight in potatoes. Therefore we hypothesized that (i) different populin applications under lab conditions may have a deleterious effect on late blight sporangia germinations, and (ii) populin application under field conditions may be similar to or even more efficient than frequently used synthetic fungicides. Altogether, we intend to offer a low-cost, accessible method for late blight control that may be useful in integrated pest management.

## 2. Material and Methods

### 2.1. P. infestans Isolates

The seven *P. infestans* strains used in this study were collected previously from potatoes in Hungary. Cultures were purified from contaminants on selective pea-broth agar (PBA) and their mating type was determined as described by Bakonyi et al. (2002) [18]. Pure isolates were maintained at the culture collection of Plant Protection Institute, Centre for Agricultural Research (Budapest, Hungary) until the experiments. Data available for the isolates are presented in Table 1.

### 2.2. Plant Extracts Preparation

Extracts from black poplar (*Populus nigra*), at a concentration of 1, 2, 3 and 4 *v*/*v*% were used according to EPPO (European Plant Protection Organization) Protocol (2019) [19]. This was done because of the rationale that oomycete diseases are of no consequence in poplar cultures [20]. Fresh plants materials (buds of black poplar), also called “gemma populy”, were collected in early spring from about 12 black poplar trees. These were *P. nigra* individuals situated around Sapientia Hungarian University, Faculty of Technical and Human Sciences areas (46.5206° N, 24.6189° E). The plant materials were first separately dried in a ventilated oven at 45 °C for 24 h. Twenty grams of the dried plant material were then weighed in a 100-mL Erlenmeyer to which 70 mL of hexane (purity grade 99%) were added for pre-extraction. The Erlenmeyer was placed in a sonicator bath (Branson 8210) and was sonicated at a temperature of 40 °C for 30 min. The mixture was then filtered, poured into a round-bottomed flask, and the solvent was concentrated (at about 11 mm Hg) to 5–10 mL by means of a rotary evaporator, using a water bath at 40 °C. The residue was then placed in a 30-mL vessel and the solvent left to evaporate overnight in a well-ventilated hood in order to eliminate the last traces of the solvent. Concentrations of 1, 2, 3 and 4 *v*/*v*% were prepared with distilled water in a sterilized Erlenmeyer, and kept at 4 °C until being used for laboratory experiments. Gas chromatographic analyses of all extracts were carried out, and the main component populin was identified at a 95% level or more from each extract (individual plant buds). Therefore, all poplar bud extracts from individual trees were mixed and used for any further analyses.

### 2.3. In Agaro Test of Poplar Bud Extracts on Mycelial Growth of Different Late Blight Strains

The whole lab experiment was carried out in 2016 and 2017. The seven strains of *P. infestans* were previously maintained on pea-broth agar (PBA) [21]. PBA test plates containing 1, 2, 3 and 4 *v*/*v*% of the extract from black poplar buds (here termed *populin*) were prepared by the addition of the required volume of filter sterilized (0.45 µm pore-size mixed cellulose ester filter, Whatman GmbH, Dassel, Germany), concentrated extract with 20% populin and varying volumes of sterile distilled water to a total of 67.5 mL combined volume to the medium after autoclaving. Plates containing no extract, only distillate water, were used as controls and received only 67.5 mL sterile distilled water. First, PBA glass plates were inoculated with 8-mm-diameter mycelial agar blocks excised from actively growing colonies of *P. infestans*. Altogether 100 plates were inoculated with each strain (700 as total), meaning 20 plates/strain/treatment (four treatments and control). Inoculated plates were then incubated for 14 days at 20 °C in darkness. Colony growth of *P. infestans* in each plate was measured daily in two directions (plates marked left, right, up and down and measured from left to right and up to down directions) over a 10 day period after the 14 days incubation using a digital caliper in units of mm, which were collected as quantitative data up to 55 mm (the plate diameter). The whole experiment was carried out twice with the same number of replicates and followed along each 10-day period. The parameters of the treatments were as follows:

Control-no populin + 140 g pea agar + 67.5 mL sterile distillate water.

1. *v*/*v*%-11.25 mL 20% populin + 140 g pea agar + 56.25 mL sterile distillate water.
2. *v*/*v*%-22.5 mL 20% populin + 140 g pea agar + 45 mL sterile distillate water.
3. *v*/*v*%-33.75 mL 20% populin + 140 g pea agar + 33.75 mL sterile distillate water.
4. *v*/*v*%-45 mL 20% populin + 140 g pea agar + 22.5 mL sterile distillate water.

When the inhibitory effect of 1 and 2 *v*/*v*% populin extract on the mycelial growth was low, the experiment was repeated again two more times (total of three repetitions) using only 3 and 4 *v*/*v*% extracts and water control. The experimental conditions, data assessment and number of replicates were the same as describe above.

### 2.4. In Planta Test of Poplar Bud Extracts on Different Late Blight Strains

Field application of populin treatment was carried out during a complete vegetation period in Central Transylvania (46°0159635″ N, 26°2074013″ E) in 2018. This area is dominated by potato fields in which traditional cultivating methods are applied. The experimental plot was established in a 1000-m^2^ area. First the area was surrounded by a 1.5-m plastic fence to prevent the experimental plot from unknown late blight strains present in the neighboring fields. The potato variety Carrera was used in the experiment, because it is one of the most frequently cultivated varieties and is often used in experiments dealing with foliage and tuber blight [22,23]. Strain M16 was tested for the “in plants” experiment. This was done because this strain was the only resistant strain under lab experiment. The experiment setup was 10 plants/block/stains/treatments all replicated three times. Replications and blocks were isolated at a distance of 5 m from each other using plastic fences of 1.5 m to keep them isolated from another plot of 10 plants and prevent cross-infection. Altogether five treatments were used for each strain and replicated two more times (three repetitions) as mentioned. These were as follows:

Control-no populin or synthetic fungicide, only distilled water 200 mL.

1. Infinito 687 SC (62.5 g/L fluopicolid + 625 g/L propamocarb) doze: 1.4 L/300 L water (1.9 mL/200 mL water) synthetic fungicide.
2. Valis M (60% mancozeb + 6% valifenalat) doze: 2.5 kg/300 L water (1.7 g/200 mL water) synthetic fungicide.
3. Populin 10%: 100 mL extract 20% + 100 mL water.
4. Populin 5%: 50 mL extract 20% + 150 mL water.

The late blight inoculum M16 was prepared and plant infestations made according to Bálint et al., 2014 [15]. Potato plants were sowed and irrigated in normal cultivation regimes during the year. Detailed assessments of plant health were made including both leaves and stems every day. No late blight or other infestations were detected prior to the experiment. When potato plants reached the BBCH 20–39 development stages [24], treatments were applied by spraying plants with fungicides (200 mL/10 plants) or populin (200 mL/10 plants) or water (control) (200 mL/10 plants). Spraying was made with sterile automated spraying systems used for pesticides under field conditions. First symptoms were observed after 24 h on the plants. Infection rates were assessed after another 24, 48 and 72 h. The whole procedure, including treatment and assessments, were replicated again twice during the vegetation period.

Field assessments of late blight infestation were made according to the EPPO protocol [19]. During the survey, whole plants from the three blocks (10 plants randomly selected from three blocks as 3/3/4 plants) were extracted after 24 h and removed. The level of infestation was determined on the leaves. To assess infection rate, the set of 10 plants/stains/treatments were considered 100%, and the percentage of infected plants was assessed again after 48 and 72 h for each treatment in a same way (extracting another 10 plants from three blocks as 3/4/3 plants after 48 h, and then 4/3/3 plants again after 72 h). To assess late blight severity, each plant was classified separately according to the EPPO recommendations as follows:

1. 0% = healthy (0 foliar damage).
2. 1% = up to 10 spots per plant or up to 1 leaflet in 10 attacked.
3. 10% = up to 4 leaflets in 10 affected; plants still retaining normal form.
4. 25% = nearly every leaflet with lesions but plants still retaining normal form; plot may look green though every plant affected.
5. 50% = every plant affected and about half of leaf area destroyed by blight; plot looks green, flecked with brown.

All applicable international, national, and/or institutional guidelines for the care and use of plant pathogens were followed. The article does not contain any studies with human and animal participants performed by any of the authors.

## 3. Data Analyses

The data from the lab experiments did not follow the assumption of normality; therefore, the Kruskal-Wallis test, followed by a Mann-Whitney test, was used to assess differences for each day during the 10-day assessment period. The effects of populin treatments were compared with controls using the average value of daily diameter data recorded during the 10-day growing period for 20 plates/strains/treatments, and 99% confidence intervals were used as statistically significant differences.

Because data from field experiment did not meet the assumption of normality, again the nonparametric Kruskal-Wallis test was used, followed by a Mann-Whitney test to compare the effect of populin, synthetic fungicide treatment and control. To compare infection rate and intensity, average data of 10 plants/blocks/treatments were used, and 95% confidence intervals were used as statistically significant differences. All analyses were run in R [25].

Principal Components Analyses (PCAs) were used to determine the effect of 3 and 4 *v*/*v*% populin treatments or their absence (control) under lab conditions on mycelial growth of different late blight strains. This was done by subjecting data on the relative amount of each strain’s dimensions in plates after 3 and 4 *v*/*v*% application and control. PCA was also used to test the effect of either populin, synthetic fungicide or its absence (control) as an environmental variable on late blight infection rate and intensity under experimental field conditions. Before analyses, all data were first averaged and log10 transformed, then PCA covariance analyses were run using Community Analysis Package 4 in R [25].

## 4. Results

**Figure 1:**
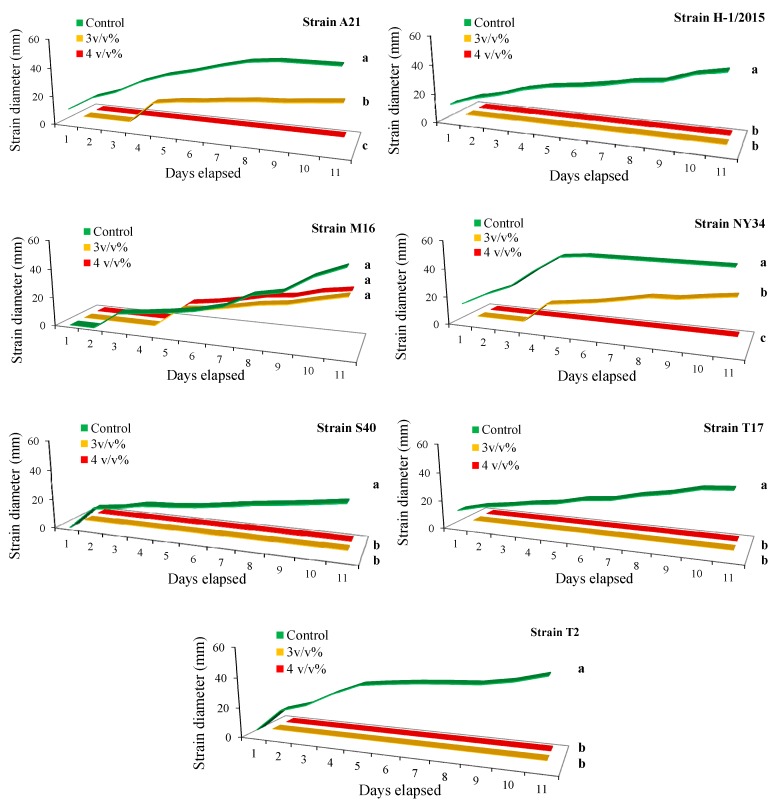
Growth (mm) of different late blight strains treated with 3 and 4 *v*/*v*% populin and distilled water (control) during the 10-day assessment period. Different letters mean statistically significant differences at the *p* ≤ 0.01 level (Mann-Whitney test).

**Figure 2:**
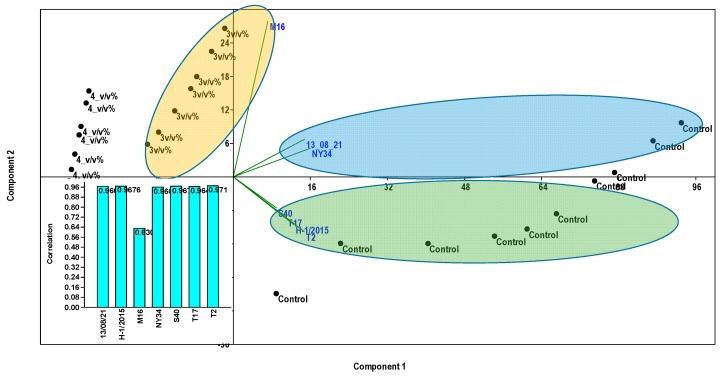
Principal Components Analyses (PCAs) showing the effects of 3 and 4 *v*/*v*% populin treatments or their absence (control) on the colony growth of different late blight strains under lab conditions. The average strain-dimension data in plates after 3 and 4 *v*/*v*% and the controls were considered and log10 transformed. Correlations between PCA values are given in bar charts. The blue and green colors represent the PCA axis direction of the control effect related to sensible stains. Yellow represents the PCA axis direction of the populin 3 and 4 *v*/*v*% concentrations effects.

Considering the average growth rate of *P. infestans* hyphae under lab conditions in the control, the most intensive growth was detected for strain NY34 (average of 46.3 mm), reaching the maximum area of 55 mm on the 5th day. This was followed by strains S40 (mean of 43.6 mm), T17 (mean of 41.4 mm), T2 (mean of 41.6 mm), H-1/2005 (mean of 36.3 mm) and M16 (mean of 33.3 mm). The lowest growth rate was detected for strain 13/08/21 (mean of 28.7 mm), which did not reach the maximum size of the plate even at day 10. By comparing the growth rate under different populin treatments and their controls, it could be detected that the 1 *v*/*v*% populin did not affect the growth of strains 13/08/21 (control 28.78 mm - 1 *v*/*v*% 20.91 mm, U = 0.7, *p* < 0.65), M16 (control 33.36 mm - 1 *v*/*v*% 32.34 mm, U = 0.9, *p* < 0.89) and T17 (control 41.45 mm - 1 *v*/*v*% 34.4 mm, U = 0.5, *p* < 0.43), but had a moderate effect on other strains after the first days and reduced the growth of mycelium significantly until the end of the experiment (Table 2). Populin treatment at 2 *v*/*v*% also had no effect on 13/08/21 (control 28.78 mm - 2 *v*/*v*% 18.28 mm, U = 0.1, *p* < 0.13) and M16 (control 33.36 mm - 2 *v*/*v*% 23.73 mm, U = 0.4, *p* < 0.32), while the 3 *v*/*v*% concentration reduced the growth rate of all strains (Table 2). Populin treatment at 4 *v*/*v*% had a clear inhibitory effect on almost all strains during the whole experimental period, except for M16 and T2, in which growth was delayed (M16 and T2 started to grow on the 5th and 7th days, respectively) and small colonies with an average of 11 to 32 mm were observed at the end of day 10 (Table 2). Running the experiment for 20 more days with 3 and 4 *v*/*v*% populin treatments revealed some interesting information about the possible resistance of late blight strains against populin. Altogether it could be observed that the 3 *v*/*v*% concentration completely inhibited the development of strains H-1/2015 (U = 3.2, *p* < 0.01), S40 (U = 3.8, *p* < 0.01), T17 (U = 4.1, *p* < 0.01) and T2 (U = 3.8, *p* < 0.01) compared with control (Figure 1). Delayed (from day 3 or 4) development of mycelium was detected in strains 13/08/21, M16 and NY34 (Figure 1). The 4 *v*/*v*% populin concentration was proved to be the most effective treatment, and its inhibitory effect was detected in all strains except for M16 (Figure 1). PCA revealed that strain M16 was less affected by populin treatments, while any other strains were substantially influenced by both 3 and 4 *v*/*v*%. Significant (rho > 0.9) positive correlation values between PCA scores of treatments and late blight colony dimensions were detected for all strains except for M16 (rho < 0.62) (Figure 2).

**Figure 3:**
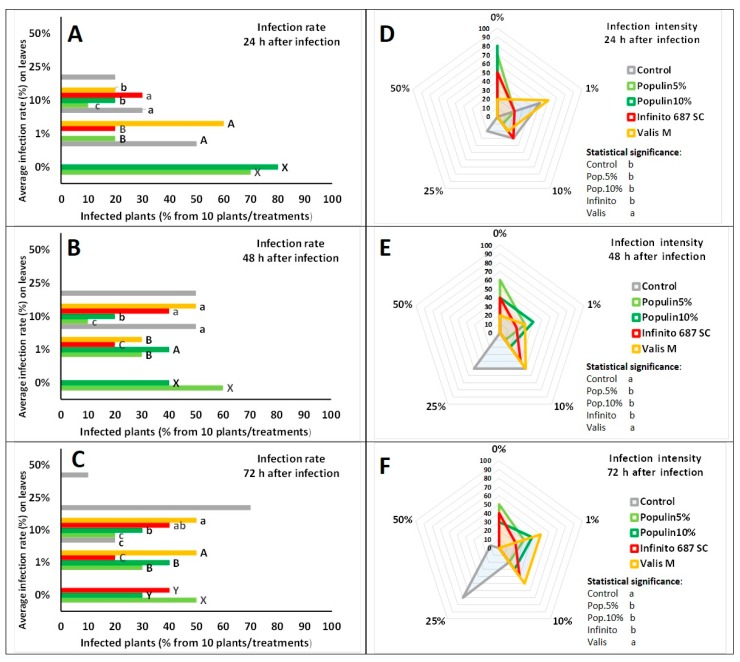
Infection rate after 24 (**A**), 48 (**B**) and 72 h (**C**), and infection intensity after 24 (**D**), 48 (**E**) and 72 h (**F**) of late blight strain M16 under field conditions. Different letters mean statistically significant differences between treatments at the *p* ≤ 0.01 level (Mann-Whitney test).

Considering assessment of the field experiment, 0% infection rate was detected after 24 and 48 h when populin 5 and 10% were applied (Figure 3A,B). Inhibitory effects due to the fungicide Infinito 687 SC was only detected 72 h after inoculation (Figure 3C). Up to 10% infection rate was detected after 48 and 72 h using populin 5% and 10% treatments, and altogether analyses revealed that no high (i.e., 25%) infection rate was detected when populin was used. The effectiveness of populin was significantly better than both Valis M and Infinito 687 SC, and the rate of infected plants with late blight were significant (80% lower) after populin treatment (Figure 3A–C). Comparing populin 5% and 10% concentrations after 24 h, the 5% concentration proved to be more effective, resulting in a very low infection rate. The effectiveness of 10% increased after 28 and 72 h (Figure 3A–C).

**Figure 4:**
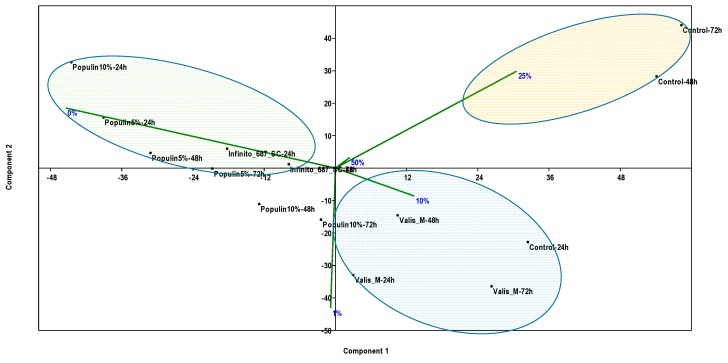
Principal Components Analyses (PCAs) showing the effect of either populin, synthetic fungicide or its absences (control) as environmental variables to late blight strain M16 infection rate under field conditions. The green color represents the PCA axis direction of the populin effect, the blue color represents PCA the axis direction of the Valis effects and the yellow color represents the PCA axis direction of the control effects.

Higher infection intensity at the 1% level was observed after 24 h using Valis M (Figure 3D). The same tendency toward 10% infection remained after 48 and 72 h using Valis M with similar high infection intensity for the control as well (Figure 3E,F). PCA also revealed the effectiveness of populin 5% and 10% under field conditions. According to PCA, synthetic fungicide Valis M allowed an average of 10% infection after 24, 48 and 72 h, while the lack of any treatments resulted in 25% infection in all time points (Figure 4). 

## 5. Discussion

Our aim was to test the effect of poplar bud extract on the potato late blight pathogen *P. infestans*. The extract considerably reduced the hyphal growth of six isolates out of seven in culture, and was significantly more effective against leaf blight in the field experiment than the two synthetic fungicides used in late blight control. Because all of the major diseases of *Populus* species are caused by true fungi, it is possible that *Populus* species (including cultivated varieties such as *P. nigra cv. italic*) generally produce compounds as populin that inhibit oomycete pathogens. Based on this, it can be concluded that the disease severity may be significantly reduced by the use of populin extract in a 5% or 10% concentration. It is also a less expensive yet available natural material that could replace fungicides under field conditions. Strain M16, however, seems to have developed resistance against populin, and the background of this resistance needs further assessment. For many growers, the repeated application of synthetic fungicides throughout the growing season has been the only available approach to managing late blight. Therefore, resistance against these fungicides now exists in many of the potato growing areas throughout the world [6,7,8]. According to our results, an application of 10% populin, one of the most accessible extracts, can therefore be easily prepared by growers at low cost. Similar experiments in our previous study demonstrated that populin extract considerably reduced the germination of apple scab (*Venturia inaequalis*) conidia, and that infestation levels furthermore were lower than in cases of conventional treatments [15].

Compounds of plant extracts may have direct inhibitory effects on plant pathogens (e.g., by fungicidal or fungistatic activities), or they can help in the establishment of favorable conditions for antagonistic microbes [26]. Extracts of several plant species have been reported to be effective against *P. infestans* in laboratory bioassays [27,28]. Wang et al. achieved 90% inhibition of some diseases including late blight in potatoes via a foliar application of 1% leaf extracts of plant extracts (*Galla chinensis*, *Potentilla erecta*, *Rheum rhabarbarum*, *Salviae officinalis*, *Sophora flavescens* and *Terminalia chebula*) [29]. In the study of Majeed et al. foliar sprays of 25% leaf extracts of three medicinal plants (*Podophyllum hexandrum*, *Withania somnifera* and *Xanthium strumarium*) at a 3-day interval significantly reduced disease severity and resulted in a higher tuber yield [30]. According to the authors, the positive effects could be attributed to the inhibitory effects of bioactive compounds on mycelial growth of *P. infestans*. Unfortunately, little is known about the biological mechanisms leading to the inhibitory effect of populin [15,16]. Since our in vitro tests have revealed a reduction of culture growth in most *P. infestans* isolates, direct inhibition of hyphal growth might be a possible mode of action. However, there must be other mechanism(s) too, because disease severity caused by the isolate which was insensitive to populin in vitro was greatly reduced in the field trials. Germination of sporangia and/or encysted zoospores could have been inhibited by populin, but we cannot exclude the role of antagonistic organisms in the phyllosphere either. Further investigations are needed to answer these questions. According to our results, including those of the apple scab [15,16,17], we conclude that controlling many severe oomycetes diseases with populin may enable the partial elimination or at least reduction of synthetic fungicide usage, thereby enabling efficient organic and/or integrated farming in regions of traditional agriculture.

## Figures and Tables

**Table 1:** Data of the *Phytophthora infestans* isolates used in this study.

Strain	Year of Isolation	Location	Mating Type	Coordinates
13/08/21	2013	Érd (HU)	Unknown	Unknown
H-1/2015	2015	Érd (HU)	Unknown	Unknown
M16	Unknown	Monorierdő (HU)	A1	N47 18.772, E19 29.118
NY34	2006	Nagykálló (HU)	A1	Unknown
S40	2006	Solt (HU)	A1	N46 46.867, E19 02.621
T17	2010	Tordas (HU)	A1	N47 20.448, E18 45.604
T2	2010	Tordas (HU)	Unknown	N47 19.817, E18 45.707

**Table 2:** Growth (mm) of different late blight strains treated with 1, 2, 3 and 4 *v*/*v*% populin and distilled water (control) during the 10-day assessment period for 20 plates/strains/treatments.

	Assessment Periods (Days)										Mean	*
	1	2	3	4	5	6	7	8	9	10		
	Growth (mm) of strain 13/08/21											
Control	11.1	14	19.1	22.6	27.8	32.6	37.8	40.9	40.9	41	28.78	**A**
1 *v*/*v*%	3.7	12.7	17.3	18.8	22.4	24.7	24.9	26.7	28.6	29.3	20.91	**A**
2 *v*/*v*%	0	0	16	17.9	20.9	22.7	24.2	25.4	27.1	28.6	18.28	**A**
3 *v*/*v*%	0	0	0	0	11.3	12.3	14	14	14	14.1	7.97	**B**
4 *v*/*v*%	0	0	0	0	0	0	0	0	0	0	0	**C**
	**Growth (mm) of strain H-1/2015**											
Control	12.7	18.5	23.8	27.3	33.3	39.5	43.3	55	55	55	36.34	**A**
1 *v*/*v*%	10.7	13.8	17.3	20	22.8	24.1	27.5	29.6	29.8	31.6	22.72	**B**
2 *v*/*v*%	10.1	11.3	13	15	16.8	17.3	17.6	20.1	20.1	20.8	16.21	**C**
3 *v*/*v*%	0	0	9.4	10.1	11.8	12.1	11.9	12.4	13	13.3	9.4	**D**
4 *v*/*v*%	0	0	0	0	0	0	0	0	0	0	0	**E**
	**Growth (mm) of strain M16**											
Control	3.2	11.1	17.2	22.2	31.3	38.6	45	55	55	55	33.36	**A**
1 *v*/*v*%	0	11.4	18.3	22.5	28.1	35.6	42.5	55	55	55	32.34	**A**
2 *v*/*v*%	0	0	15	16.3	20.5	26	30.7	37.5	45.1	46.2	23.73	**AB**
3 *v*/*v*%	0	0	11.6	15.7	15.7	18.4	21.4	27.3	28.6	32.5	17.12	**BC**
4 *v*/*v*%	0	0	0	0	16.5	19.8	21.7	23.9	29.2	32.5	14.36	**C**
	**Growth (mm) of strain NY34**											
Control	17.2	29.5	38.2	48.6	55	55	55	55	55	55	46.35	**A**
1 *v*/*v*%	13.3	18.2	23	25.5	29.4	34.3	38.2	44.6	47.9	48.9	32.33	**B**
2 *v*/*v*%	0	0	15	16.8	18.3	20.3	22.8	24.5	25.1	27.8	17.06	**C**
3 *v*/*v*%	0	0	12	12.9	12.9	14.7	17.2	19.1	19.3	20.2	12.83	**D**
4 *v*/*v*%	0	0	0	0	0	0	0	0	0	0	0	**E**
	**Growth (mm) of strain S40**											
Control	14	24.8	32.5	39.9	50.4	55	55	55	55	55	43.66	**A**
1 *v*/*v*%	4.5	13	21.2	24.7	30.5	35.5	39.7	43.5	44.2	47.6	30.44	**B**
2 *v*/*v*%	0	0	13.8	15.8	20.5	25.3	29.2	33.6	36.8	43	21.8	**C**
3 *v*/*v*%	0	0	0	0	11.2	12.7	12.9	15.1	15.8	15.9	8.36	**D**
4 *v*/*v*%	0	0	0	0	0	0	0	0	0	0	0	**E**
	**Growth (mm) of strain T17**											
Control	13.6	19.1	28.1	34.8	43.9	55	55	55	55	55	41.45	**A**
1 *v*/*v*%	11.3	16.8	20.9	25.2	29.6	35.5	39.7	55	55	55	34.4	**A**
2 *v*/*v*%	0	0	16.1	19.2	24.2	27.4	29.2	33.3	38.8	46.4	23.46	**B**
3 *v*/*v*%	0	0	10.5	11.6	13.8	15.5	15.9	17	20.1	20.9	12.53	**C**
4 *v*/*v*%	0	0	0	0	0	0	0	0	0	0	0	**D**
	**Growth (mm) of strain T2**											
Control	17	22.8	29	33.8	42.8	51	55	55	55	55	41.64	**A**
1 *v*/*v*%	13	15.8	19.8	22	24.7	27.4	29	30.4	32.1	33.3	24.75	**B**
2 *v*/*v*%	11.8	13.7	16.9	19.1	21.8	24	25.1	27.4	29.2	30.9	21.99	**B**
3 *v*/*v*%	0	0	11.4	12.2	13.5	14.2	15.7	15.7	15.7	15.7	11.41	**C**
4 *v*/*v*%	0	0	0	0	0	0	10.7	11.3	11.3	11.3	4.46	**D**

* Significance: Means with different letters are significantly different from each other according to the applied statistical test (*p* ≤ 0.01, Mann-Whitney test).
